# A cross-sectional online survey of depression symptoms among New Zealand’s Asian community in the first 10 months of the COVID-19 pandemic

**DOI:** 10.1080/03036758.2023.2251900

**Published:** 2023-09-03

**Authors:** Richard J. Siegert, Andrew Zhu, Xiaoyun Jia, Guanyu Jason Ran, Nigel French, David Johnston, Jun Lu, Liangni Sally Liu

**Affiliations:** a Department of Psychology & Neuroscience, School of Clinical Sciences, Auckland University of Technology, Auckland, New Zealand; b Trace Research Ltd, Auckland, New Zealand; c Institute of Governance & School of Political Science and Public Administration, Shandong University, Qingdao, People’s Republic of China; d School of Applied Sciences, Edinburgh Napier University, Edinburgh, Scotland; e Infectious Diseases Research Centre, Hopkirk Research Institute, Massey University, Palmerston North, New Zealand; f Joint Centre for Disaster Research, Massey University, Wellington, New Zealand; g Auckland Bioengineering Institute, University of Auckland, Auckland, New Zealand; h School of Humanities, Media and Creative Communication, Massey University, Auckland, New Zealand

**Keywords:** Depression, Asian, CES-D, racism, COVID-19, factor analysis, network analysis

## Abstract

The COVID-19 pandemic has elevated levels of distress and resulted in anti-Asian discrimination in many countries. We aimed to determine the 10-month prevalence of depression symptoms in Asian adults in New Zealand during the pandemic and to see if this was related to experience of racism. An online survey was conducted and a stratified sample of 402 respondents completed the brief Centre for Epidemiological Studies-Depression (CES-D) scale. Analyses included: descriptive statistics, depression scores by age/gender, factor analysis of the 10 item CES-D and partial correlation network analysis of CES-D items together with questions about experience of racism. Results show that half of the sample reported clinically significant symptoms of depression. Depression was higher among younger participants but there was no gender difference. Internal consistency was high (α = 0.85) for the CES-D which revealed a clear two-factor structure. Network analysis suggested that sleeping problems might be the bridge between experiences of racism and depression. The prevalence of low mood was high with clinically significant levels of depressive symptoms. Depression was higher in younger people and had a modest positive correlation with personal experience of racism.

## Introduction

The COVID-19 pandemic has increased stress and the prevalence of some mental disorders–at least where this has been researched. A systematic review in 2020, noted high levels of anxiety, depression, post-traumatic stress disorder and stress among the general population in China, Spain, Italy, Iran, the US, Turkey, Nepal, and Denmark, and these levels were generally higher than pre-pandemic rates (Xiong et al. 2020; De et al. 2021). Similarly, in New Zealand, there is evidence which suggests that its adult population experienced additional stressors impacting on psychological wellbeing. For example, Every-Palmer et al. (2020) used a commercial survey platform to examine the mental health of a demographically representative sample of 2,010 adult New Zealanders in April 2020. They found 30% of respondents reported moderate or severe psychological distress, 16% moderate or high anxiety, and 39% low wellbeing. Of particular concern 6% reported suicidal ideation, 2% reported suicidal plans and 2% suicide attempts.

Among all indicators of mental health, levels of depression and anxiety are two metrics that have been frequently examined to assess people’s mental wellbeing during the pandemic (Murata et al. 2021; Shevlin et al. 2020; Wu et al. 2021; Zavlis et al. 2021). For example, Shevlin et al. (2020) found a ‘modest increase’ (p. 8) in the prevalence of mental health symptoms in a stratified sample of 2,025 UK adults during the early days of the pandemic (March 2020)–with increases in anxiety, depression and trauma symptoms compared to three previous general population surveys. In New Zealand, based on an online survey of 681 adults in May-June 2020, Gasteiger et al. (2021) found that levels of depression and anxiety were significantly higher than (pre-pandemic) population norms during the pandemic. However, there is almost no research that focuses on the mental health of one significant ethnic group in New Zealand–i.e. the Asian population, the second largest, fastest growing, and most diverse non-European ethnic group. Of the limited research which focuses on this substantial ethnic minority, an online survey of 663 Asian New Zealanders in April–May 2021, commissioned by an Asian mental health service provider, reported 44% of participants reported symptoms of depression, with younger people more at risk and females only slightly more at risk than males (Zhu et al. 2021). However, there has been no study to date that disaggregates this population into different ethnic sub-groups to understand if the pandemic impacts on their depression levels differently or which variables might influence this.

Existing studies suggest that apart from major demographic variables (including younger age, being female, being unemployed/a student, urban dwelling, and having other health conditions) which predicted anxiety or depression during the pandemic (Shevlin et al. 2020)–having limited social and psychological resources, high exposure to social media and/or COVID-19 news, and experiencing racism are also detrimental to physical and mental health, especially mental health (Fitzpatrick et al. 2020; Paradies et al. 2015; Shi et al. 2022; Talamaivao et al. 2020). A study of seven countries in Asia during the pandemic found risk factors for psychological distress included age < 30, higher education, being single or separated, discrimination by other countries and contact with people with the COVID-19 virus. Protective factors included male gender, larger households, employment, confidence in doctors and spending less time viewing health information (Wang et al. 2021b). Wang et al. (2021a) compared mental health, as indexed by scores on the Depression, Anxiety and Stress Scale (DASS-21), in 4612 participants from eight countries using a chain mediation model. They found that physical health symptoms similar to those of COVID-19 were associated with higher levels of anxiety, depression and stress–but this relationship was mediated by both the individual’s need for health information and their perceived impact of the pandemic.

Some research suggests that racial discrimination Asians have experienced is positively associated with depressive symptoms during the pandemic (Lee et al. 2022; Woo and Jun 2021). It is worth mentioning that of the Asian population in New Zealand, 77% were born overseas, which means that a large majority of New Zealand Asian people are also immigrants (Stats NZ 2018). This immigrant status together with their Asian ethnicity and the recent pandemic’s political climate of growing hostility toward Asians, implies a potential risk of experiencing racism for this population during the pandemic (Liu et al. 2022), which could negatively impact on their mental health.

Based on an online survey, the present article aims to fill a knowledge gap around the prevalence of symptoms of depression in the New Zealand Asian community during the first ten months of the COVID-19 pandemic, and to clarify which variables are associated with higher symptom levels. Specifically, we examine the relationship between demographic variables (age, gender, geographical location, and ethnicity), experience of racial discrimination/stigmatisation and symptoms of depression. We included questions concerning racism after media reports that the pandemic was resulting in an increase in bullying and harassment of Chinese and other Asian people (Foon 2020). We focused on the first ten months of the emerging pandemic. This period was the time when many uncertainties were still surrounding COVID-19, vaccines were unavailable, and New Zealand experienced a series of strict lockdowns; hence our focus on monitoring symptoms of anxiety and depression.

The significance of this paper is that it is the first study which focuses on depression symptoms among different Asian groups within New Zealand and systematically investigates variables that might influence depression. Quite often, public health statistics in New Zealand group diverse Asian groups together as one single ‘Asian’ ethnic group without recognising the inherent heterogeneity and diversity, this research challenges this arbitrary categorisation of this ethnic population and presents evidence-based information about the prevalence of depression of this important ethnic group in New Zealand during the pandemic to aid public health decision making and interventions. The result of this research is timely because Asians have been historically and consistently overlooked in the country’s health sector. The last comprehensive *Asian health report* funded by the Ministry of Health was 16 years ago, and the most recent analysis of Asian health data from the annual *New Zealand Health Survey* was completed six years ago. Most recently, in the latest planning report of New Zealand’s health sector transformation–*Te Whatu Ora* has also failed to address Asian health needs (Xia 2022).

## Methods

### Sampling and data collection

An online survey was used to collect data. Data were collected between the 5th and 18th of December 2020. A sample of 1,101 participants were randomly selected from the Asian Research Panel (N = 32,000) of Trace Research the only Asian research panel of its kind in New Zealand (https://www.traceresearch.co.nz/). An e-mail invitation to the 1,101 participants randomly selected for the survey was sent and the total response rate was 402 (36.5%) when all quotas were filled. Thus, a sample of the Asian adult population, stratified by age, ethnicity, gender and geographical location, according to the 2018 New Zealand Census, was used to ensure representativeness of all Asian ethnic groups in the country. The researched subject, Asians, were identified by their countries of origin. Both overseas and New Zealand-born Asians were included in the sampling. Only complete questionnaires without missing answers could be submitted for analysis. Each respondent was allowed to submit the questionnaire once only, according to the IP address recorded by the research panels.

The margin of error (MOE) is ± 4% at the 95% level of confidence. The margin of error is a statistic expressing the amount of random sampling error in the results of a survey. The larger the margin of error, the less confidence one should have that a poll result would reflect the result of a census of the entire population. In order to achieve MOE at ±4% at the confidence level of 95%, 306 or more responses are needed, to achieve ±3%, 544 responses are needed. Due to time and budget constraints, we chose the ±4% option.

### Participants

Participants were 402 Asian adults from 17 geographical regions in New Zealand recruited from a New Zealand based Asian research panel. The sample comprised 197 (49%) males, 203 (50.5%) females and two who preferred not to state their gender (0.5%). Age groups were as follows 18–29 (n = 134, 33.4%) 30–49 (n = 141, 35%), 50–64 (n = 94, 23.4%) and 65 + years (n = 33, 8%). There were 31 New Zealand-born Asians, comprising 4.2% of the total sample. Apart from these 31 New Zealand-born participants, the country of origin of the remaining 371 participants was as follows: India (33.6%), China (31.3%), Philippines (10.3%), Korea (5%), Malaysia (4.7%), Japan (2.5%), Sri Lanka (2.3%), Singapore (2.1%), Hong Kong (2.0%) and the remaining 6.2% from Taiwan, Vietnam, Cambodia, Thailand, and other Asian countries. Full demographic details of the sample are reported in Table 1.

**Table 1.** Demographic characteristics of the sample (N = 402).

Variable	N	%
**Gender** Male Female Other	197 203 2	49.0% 50.5% 0.5%
**Age Group** 18–29 years 30–49 years 50–64 years 65 + years	134 141 94 33	33.4% 35.1% 23.4% 8.1%
**Employment status** Full-time Part-time Retired Student Unemployed Homemaker Prefer not to say	238 53 31 23 24 28 5	59.3% 13.1% 7.7% 5.8% 6.0% 6.9% 1.3%
**Income per annum** $15,000 or less $15,001–$25,000 $25,001–$30,000 $30,001–$40,000 $40,001–$50,000 $50,001–$70,000 $70,001–$100,000 $100,001–$150,000 $150,001–$200,000 $200,001 + Prefer not to say	35 27 26 27 38 84 53 31 9 7 62	8.7% 6.6% 6.6% 6.6% 9.5% 20.9% 13.1% 7.7 2.2 1.8 15.5
**Household situation** Young, single, lives alone Group, flatting Young couple, no children Family with mainly preschoolers Family with mainly school aged children Family with mainly adults Older couple/single person no children at home Prefer not to say	23 53 58 51 74 84 45 13	5.8% 13.3% 14.4% 12.7% 18.8% 21.0% 11.3% 3.1%
**Region currently residing in** Northland Auckland Waikato (Hamilton) Waikato (other) Bay of Plenty Hawke’s Bay Taranaki Manawatu/Wanganui Wellington Tasman Marlborough Canterbury (Christchurch) Canterbury (other) Otago (Dunedin) Otago (Queenstown) Otago (other) Southland	6 262 15 1 6 3 4 9 52 1 2 28 2 3 2 1 4	1.6% 65.1% 3.8% 0 .2% 1.6% 0.7% 1.1% 2.2% 12.8% 0.3% 0.5% 7.0% 0.5% 0.8% 0.5% 0.3% 1.0%

### Measures

*The Centre for Epidemiological Studies–Depression scale short version (CES-D10)*: The CES-D10 is a ten-item self-report questionnaire developed to detect depressive symptoms in large epidemiological studies. The CES-D10 asks about the frequency of 10 symptoms of depression, with participants responding on a four-point Likert scale from 0 = *rarely or not at all* to 3 = *all the time*. Scores can range from 0–30, with higher scores representing greater levels of depressive symptoms. Scores ≥10 are taken to represent clinically significant levels of depression (Andresen et al. 1994).

*Racism and discrimination questions*: The CES-D10 was included as part of a broader study on the experiences of the New Zealand Asian community in the first year of the pandemic. To measure the existence and level of pandemic-driven racism and discrimination, four questions were developed by the research team and asked based on the distance of the respondents’ social ties, ranging from oneself to significant others, to media environment, and the public perception in general: (1) Since the COVID-19 outbreak in New Zealand, have you been discriminated against (e.g. making offensive remarks about your race, verbal/physical abuse) because of your ethnicity? (2) Do you know people in your immediate social environment who have encountered racist comments and/or discrimination against your ethnicity during the COVID-19 pandemic? (3) Have you noticed any racist comments against your ethnicity in the media/social media? And (4) Have you noticed any stigmatisation associated with COVID-19 during the pandemic? Each question was answered in three categories: ‘yes’, ‘no’, and ‘not sure’, and frequency analyses were performed.

### Statistical analyses

All statistical analyses were completed using IBM SPSS Statistics 27.0 except for the network analysis which used JASP version 0.14.1 (Wagenmakers 2020). We completed the following analyses:

1. Descriptive statistics for the 10 CES-D items and total score.
2. Compared age, gender, ethnic sub-group, and geographical location CES-D total scores.
3. Reliability estimated by raw Cronbach’s α and construct validity by exploratory factor analysis of CES-D items using principal component analysis and Varimax rotation. In addition, we calculated McDonald’s Ω (omega) as there has been criticism of α despite its almost universal use (Tavakoli and Dennick 2011; Hayes and Coutts 2020).
4. Partial correlation network analysis: We included all the CES-D10 symptoms in the network analysis and the four survey questions concerning experience of racism.

Network analysis of partial correlation relationships among the ten CES-D10 items and four questions concerning experience of racism using EBICglasso was conducted. LASSO with EBIC controls the number of connections through a tuning factor gamma set at 0.5 (Foygel and Drton 2010). For network visualisation the ‘Spring’ layout was adopted. ‘Spring’ places nodes with more and stronger associations more centrally in the network (Fruchterman and Reingold 1991). Centrality measures provide further information on network nodes, such as ‘betweenness’ (how often a node acts as a connecting point based on the number of paths through that node to other nodes), ‘closeness’ (how close a node is to other nodes using the average weight of the paths from that node) and ‘degree’ (the sum of all weights from that node or strength) (Montazeri et al. 2020). We tested the accuracy of the network (edges and centrality indices) using a bootstrapping procedure that measures how the correlation between the original network value and the bootstrapped sample value decreases with smaller samples.

## Results

### Descriptive statistics for items and total CES-D

**Figure 1.**
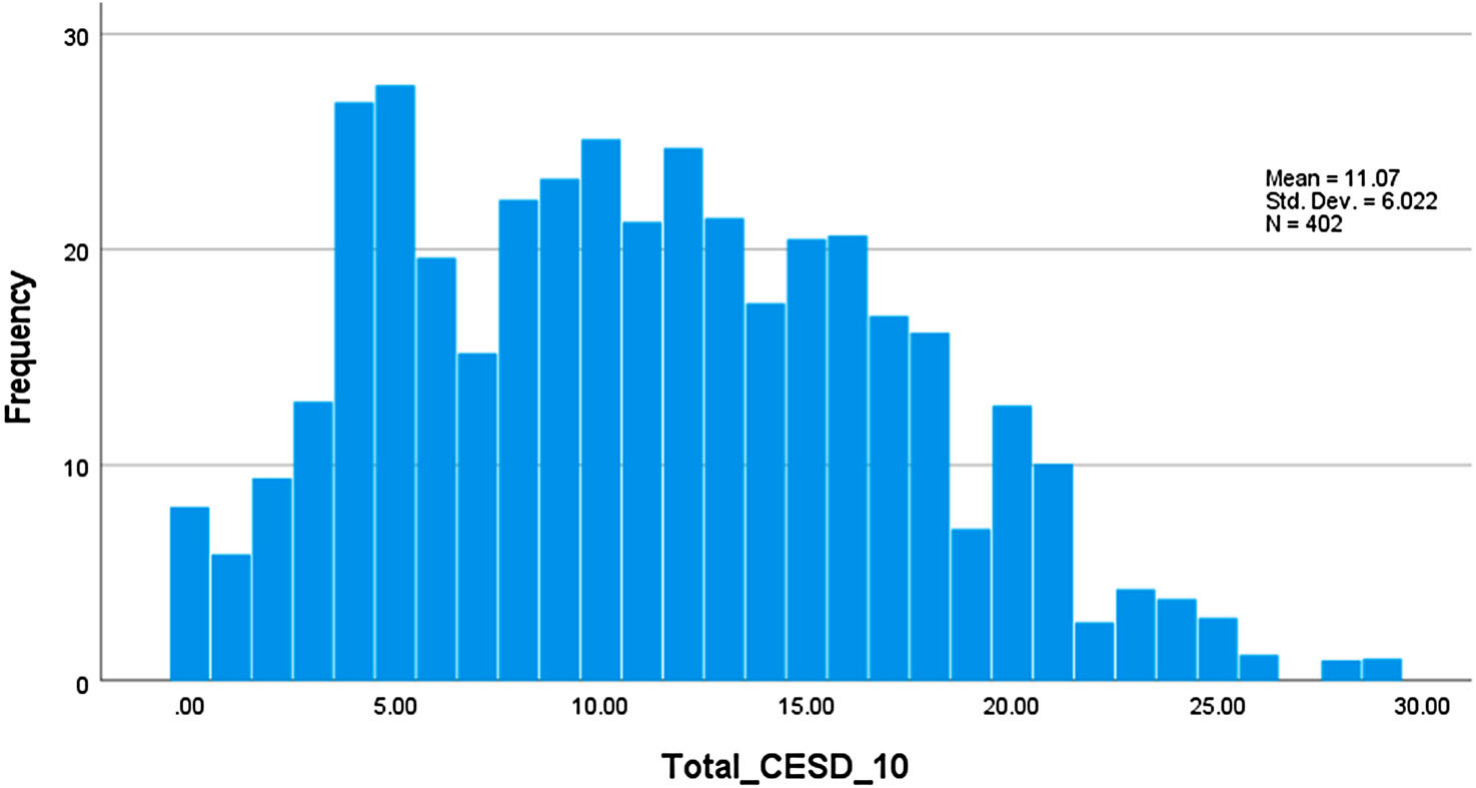
Distribution of CES-D10 total score (n = 402).

Table 2 presents the range, mean, median, mode and standard deviation for the 10 CES-D items. Participants used the full response range (0–3) for all 10 items. The items with higher means reflect those items that are more frequently endorsed (i.e. score higher) and hence the most frequent or common symptoms. As can be seen in Figure 1, the total CES-D score ranged from 0 to 29 with a mean = 11.07 and standard deviation = 6.02. The figure presents the distribution of total scores for the sample of 402. Complete percentile frequencies are reported in the Online Supplementary Data.

**Table 2.** Results of CES-D symptoms ranked highest to lowest.

Items (n = 402)	Range	Mean	Median	Mode	S.D.
8. I was happy*	0–3	1.34	1	1	0.88
4. I felt that everything I did was an effort	0–3	1.24	1	1	0.99
1. I was bothered by things that usually don't bother me	0–3	1.19	1	1	0.93
6. I felt fearful	0–3	1.09	1	1	0.88
7. My sleep was restless	0–3	1.06	1	1	0.92
2. I had trouble keeping my mind on what I was doing	0–3	1.05	1	1	0.93
9. I felt lonely	0–3	1.04	1	1	0.93
3. I felt depressed	0–3	1.04	1	1	0.91
5. I felt hopeful about the future*	0–3	1.03	1	1	0.89
10. I could not ‘get going’	0–3	0.99	1	1	0.90

* = Positively worded items that were rescored.

Total CES-D scores ranged from 0 to 29 with a mean of 11.07 (SD = 6.02). median 11, mode 5 and a range of 29. Quartile cut-offs fell at 6 (25%), 11 (50%) and 15.6 (75%).

### Gender, age, ethnic sub-group and geographical location CES-D scores

*Gender.* The means for males and females were 11.08 (6.06) and 11.05 (6.01) respectively and this was not significantly different (t = 0.06, df = 398, *p* = .95). Within the four age groups the only significant gender difference for total CES-D score was for the 30–49 year olds (male Mean = 11.91 (6.01), female Mean = 9.70 (5.37) (t = 2.29, df = 139, *p* < 05)). However, the difference for the youngest age group might be described as ‘approaching significance’ (male Mean = 12.02 (4.89), female Mean = 13.7 (5.97) (t = −1.78, df = 130, *p* = 0.08)).

**Figure 2.**
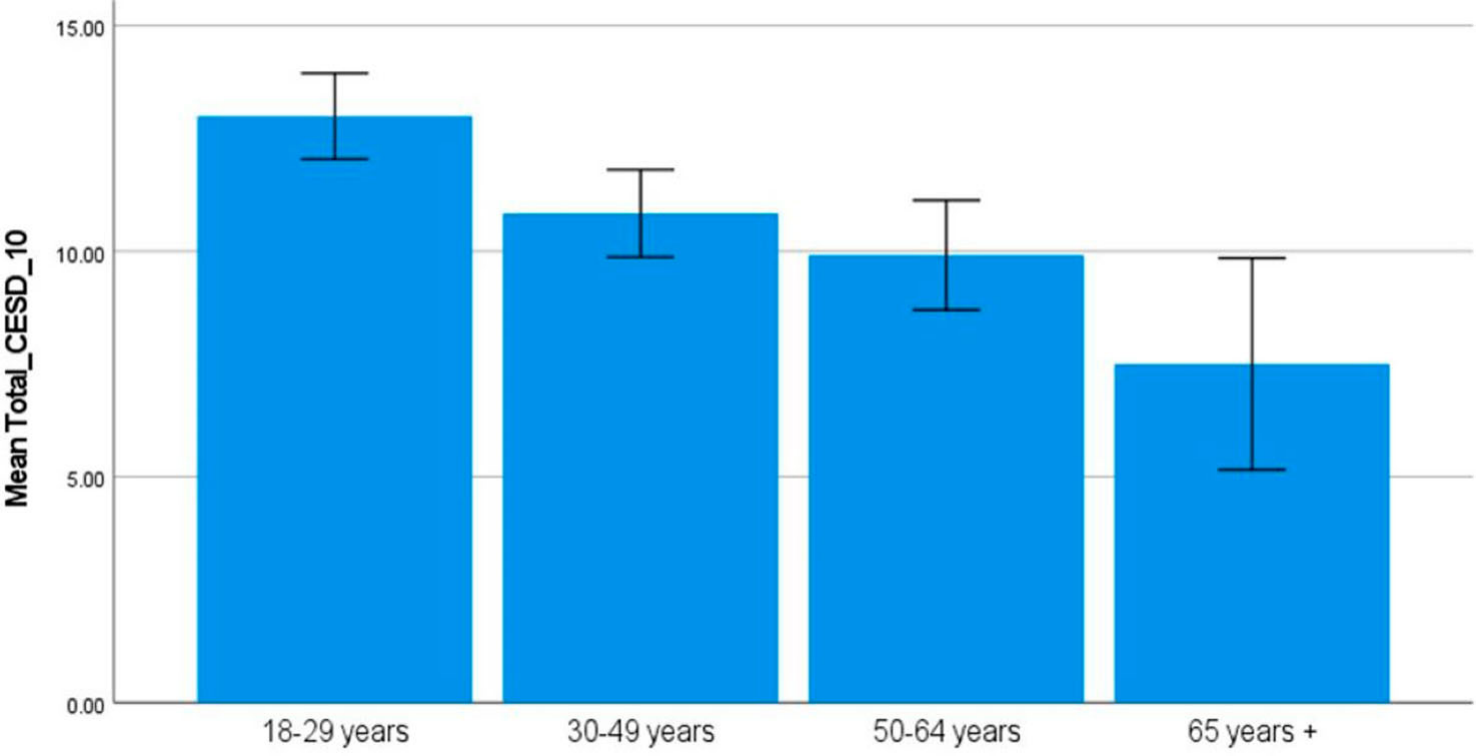
Means for 10 item CES-D total score by Age Group.

*Age.* The mean CES-D showed a stepwise decrease over the four age groups with younger participants showing higher scores (i.e. lower mood). This trend was significant according to a one-way ANOVA (F = 10.26, df = 3, *p* < 0.01). Post-hoc Tukey tests with a Bonferroni correction showed no significant difference between the 30–49 year-old and the 50–64 year-old, nor for the 50–64 year-old and the 65 + age group (Figure 2).

*Ethnic sub-groups.* It was not possible to compare total CES-D scores for all 11 ethnic groups due to the small number of participants in most of these. Consequently, we only report the mean here for the four ethnic groups with at least 20 participants (in ascending order): Philippines (M = 9.37, SD = 5.88, n = 41), Mainland China (M = 10.05, SD = 4.62, n = 126), India (M = 12.60, SD = 6.47, n = 135), and Korea (M = 12.75, SD = 5.24, n = 20). Full results (N, Mean, SD) are available for each of the 11 ethnic groups in the online supplement.

*Geographical location.* It was not possible to compare CES-D scores for the 17 geographical locations since 77% of participants lived in either of two urban locations (Auckland 65% and Wellington 12%). However, full details (N, Mean, SD) for each of the 17 geographical regions are available in the online supplement.

### Reliability and factor structure of CES-D

Internal consistency measured by the raw Cronbach’s alpha was high at α = 0.85 and also by McDonald’s omega (0.86). Inter-item correlations ranged from −0.02 to 0.65 and corrected item-total correlations ranged from 0.24 (item 5, I felt hopeful about the future and item 8, I was happy) to 0.74 (Item 3, I felt depressed). The Kaiser-Meyer-Olkin measure of sampling adequacy was high (KMO = .898), and Bartlett’s test of sphericity was significant (*p* < 0.01), indicating the correlation matrix was suitable for factor analysis. We extracted two components based on a parallel analysis. The first two principal components accounted for 59% of total variance (46% and 13% respectively). Item loadings on the first unrotated principal component ranged from 0.275 to 0.819. Inspection of Table 3 shows two factors which can be labelled as *positive* and *negative* mood.

**Table 3.** Rotated factor loadings.

CES-D item	Factor 1	Factor 2
2. I had trouble keeping my mind on what I was doing	.80	
1. I was bothered by things that usually don’t bother me	.79	
3. I felt depressed	.77	
10. I could not get going	.76	
9. I felt lonely	.72	
6. I felt fearful	.70	
4. I felt that everything I did was an effort	.69	
7. My sleep was restless	.69	
5. I was happy		.81
8. I felt hopeful about the future		.79

### Network analysis of CES-D10 symptoms and four racism questions

**Figure 3.**
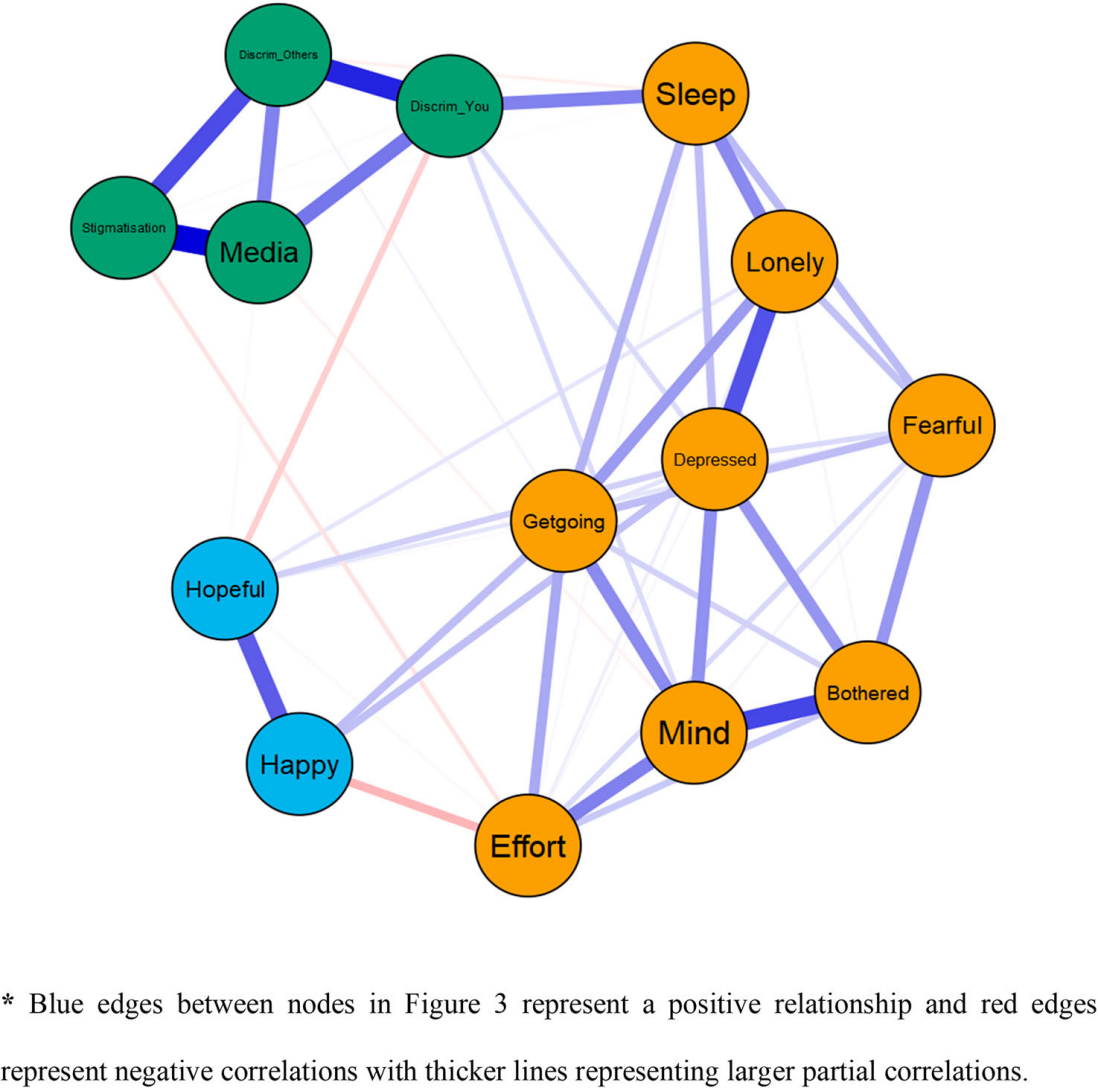
Network of 10 CES-D items and the 4 questions about recent experience of racism.

Results of the network analysis are presented in Figure 3. Bootstrapping suggested the edges between nodes were very stable with a correlation of 0.75 between the original network edges and a bootstrapped sample at just 25% of the full sample. Centrality indices were all reasonably stable with a correlation of close to 0.75 or higher at 50% of the full sample.

Blue edges between nodes in Figure 3 represent a positive relationship and red edges represent negative correlations with thicker lines representing larger partial correlations. Inspection of Figure 3 shows that the network analysis confirms the two-factor structure of the CES-D10 with the eight negative mood items clustered tightly together and similarly for the two positive mood items. The three most central or influential nodes or items were ‘I had trouble keeping my mind on what I was doing’, ‘*I felt depressed* and *I could not* “get going”’. Interestingly these three items were among the four highest loadings on Factor 1 Negative Mood. The four items about experience of racism were strongly connected to each other and linked to depression through the bridging item ‘Have you been discriminated against?’ which had a correlation with ‘My sleep was restless’.

## Discussion

The major finding in this study was a relatively high prevalence of symptoms of depression, compared with annual prevalence for the general population (see below), among a sample of 402 Asian participants living in New Zealand during the first ten months of the pandemic. However, these rates were comparable with samples in Hong Kong during the pandemic (Cheng et al. 2021). To place this finding in context the data were collected over a fortnight in December 2020 when the country was enjoying relative normality due to elimination of the first wave of the pandemic (Jefferies et al. 2020). It was the first month of summer and the entire country was in Level 1 of the New Zealand (level 1–4) alert system which involved few restrictions and New Zealand had been at this level since October 7 (New Zealand Government 2022). However, participants were specifically asked about their experience of all10 symptoms since the outbreak of COVID-19 in March 2020, with all 10 symptoms summed to yield a single global score, so these data should be interpreted as the annual (or 10 months) prevalence of mood symptoms in 2020 rather than the point prevalence in December.

We found a high prevalence of clinically significant depressive symptoms with 51.2% of the sample scoring ≥ 10 (more information is available in the supplement) which is the generally accepted CES-D10 cut-off score for clinically significant levels of symptoms. A national survey conducted in 2006 found the overall annual prevalence of major depression, as diagnosed by a structured interview, in NZ was 5.7% (Wells et al. 2006). However, it is important to note that among our sample the severity of symptoms ranges widely and includes mild, moderate and severe cases. We can compare these results with (Cheng et al. 2021) who surveyed 481 Hong Kong adults from April 20 to May 19 in 2020 during what the authors called Hong Kong’s ‘second wave’ of COVID-19 infections. They reported a mean CES-D10 score of 9.85 (SD = 2.96) and noted that ‘slightly more than half (52%) were categorised as having probable depression’ (Cheng et al. 2021, p. 5). They also found that persons higher in depression had less flexible coping strategies and greater COVID-19 related anxiety, but sex and age-cohort or ‘generation’ (Cheng et al. 2021, p. 5) (they compared Millennials, Generation X, Baby Boomers, Silent Generation) were not related to depression . In summary, we observed a high annual prevalence of symptoms of depression in our New Zealand Asian sample that was comparable with the point prevalence observed in a Hong Kong sample early in the pandemic.

In the present study there was no significant difference in the levels of depression for males and females, but younger participants reported higher rates of depression. There was a modest, significant positive correlation between depression and experiences of racism with a network analysis suggesting that direct personal experience of racism and disturbed sleep might conceivably act as a bridge or mechanism by which racism influences mood. However, this is based on a cross-sectional, correlation network and must be regarded as a hypothesis arising from our results, one that requires closer examination with a longitudinal study. At the same time, it is consistent with longitudinal studies indicating that sleep mediates the effect of perceived racism on depression among African-American and Latin American people in the United States (Steffen and Bowden 2006; Pichardo et al. 2021).

The CES-D10 proved to be a reliable measure for use with an Asian sample in New Zealand as shown by a Cronbach’s α of 0.85 which reflects high internal consistency or scale reliability. The two-factor structure (positive and negative symptoms) we observed in the present study was also quite consistent with the structure reported in studies from other countries e.g. South Africa (Baron et al. 2017). The separation into positive and negative dimensions of mood was confirmed by the network analysis which also saw these two sets of symptoms or items forming two distinct albeit connected clusters. The network analysis also identified three symptoms as the most central in the network–‘I had trouble keeping my mind on what I was doing’ (concentration), ‘I felt depressed’ (low mood) and ‘I could not *get going*’ (low energy). As the most central symptoms, these three items have the greatest number of connections with other symptoms, the strongest correlations with other symptoms and most often act as the pathway or link between pairs of unconnected symptoms. Interestingly, they formed three of the four highest loading items on the first rotated factor of Negative Mood.

While the precise interpretation of centrality measures in the application of NA to psychological networks remains the focus of some debate and uncertainty there is a good argument that more central symptoms could be the focus of assessment and intervention (Bringmann et al. 2019). Recently, Cheung and colleagues used NA to examine depressive symptoms among 11,072 Hong Kong residents during the early stages of the COVID-19 pandemic (March to April 2020) (Cheung et al. 2021). Their study used the PH9 to measure symptoms of depression and found the three most central symptoms were Guilt, Sad Mood and Energy. As the CES-D10 and the PHQ9 have quite different sets of items it is not possible to make direct comparisons. For example, the PHQ9 has items about guilt and suicide which the CES-D10 does not while the CES-D10 alone has positive mood items. Nonetheless the Sad Mood (*Feeling down, depressed or hopeless*) and Energy (*Feeling tired or having little energy*) items are similar in content to the two CES-D10 items *I felt depressed* and *I could not get going*–suggesting some degree of similarity across results. Interestingly, a systematic review of network analyses of major depression that included 254 studies of general and clinical samples and compared 58 cross-sectional networks concluded that fatigue and depressed mood were the two most central symptoms playing a critical role in or understanding of this disorder (Malgaroli et al. 2021).

Finally, the present study highlights the need to consider and plan for the mental health consequences of future pandemics in addition to their direct effects on physical health, and begs the question of what governments can do in this regard? While addressing this question is beyond the scope of the present research, our study underscores the vital need to learn from the COVID-19 pandemic and prepare for future pandemics and other catastrophic events (see Campion et al. 2022; Sachs et al. 2022). For example, there is evidence from a meta-analysis of 33 countries that those countries which implemented more stringent measures to restrict the spread of COVID-19, experienced a lower prevalence of depressive symptoms (Lee et al. 2021).

## Limitations

The present study had several limitations which need to be acknowledged. First, it is a cross-sectional study which limits the conclusions which can be drawn from it. For example, it is not clear to what extent the prevalence of depressive symptoms was unusually high due to the pandemic, or if these rates might actually be typical of the New Zealand Asian community. Moreover, we could not find well validated cut-off values to distinguish mild, moderate and severe depression using the CES-10. A longitudinal study is necessary to demonstrate that the pandemic actually produced an increase in low mood among the Asian community. Second, we asked about symptoms since the pandemic started, similar to annual prevalence, whereas most studies using the CES-D10 ask about the *past few weeks* meaning point prevalence. Hence caution needs to be exercised when comparing our prevalence rates with other studies. Third, for practical reasons of minimising participant burden, we only focused on one dimension or category of psychological distress, namely depression or low mood. It would have been informative to ask about a more diverse range of forms of distress including anxiety, alcohol/drug abuse and loneliness. It would also have added balance to inquire about some more positive aspects of wellbeing such as coping and resilience. Certainly, it would be worth exploring these avenues in the future using longitudinal studies with our Asian community. A fourth limitation, is that our sample comprised only small numbers of participants from Asian countries other than China, India, Korea and the Philippines, limiting the generalisability of our findings to other ethnic groups. Moreover, 77% of our sample was drawn from two major cities (Auckland and Wellington). While this closely reflects the geographical distribution of the total Asian population in New Zealand (see https://www.stats.govt.nz/tools/2018-census-ethnic-group-summaries/asian), it suggests that future research might focus on the experience of Asian people in rural areas with very small Asian communities. Future studies might also want to make a special effort to recruit older Asian participants as our sample of 402 only included 33 people aged 65 + .

## Conclusion

The annual prevalence of low mood was quite high with half the sample scoring above the cut-off point for clinically significant levels of depressive symptoms and this rate was comparable with a Hong Kong sample collected early in the pandemic. However, this figure aggregates mild, moderate and severe depression. Depression was higher in younger people and had a modest positive correlation with personal experience of racism–possibly due to its effect on sleep. There was no difference in severity of depression observed between males and females. The CES-D10 proved a robust tool for the measurement of mood in an Asian New Zealand sample. Longitudinal studies that examine a broader range of dimensions of distress and employ a longitudinal design are now needed.

## Supplementary Material

Supplemental material
