# Supplementary material for: A cross-sectional online survey of depression symptoms among New Zealand’s Asian community in the first 10 months of the COVID-19 pandemic
Source: J R Soc N Z. 2023 Sep 3;55(1):98–112. doi: 10.1080/03036758.2023.2251900 (PMC11619012; doi:10.1080/03036758.2023.2251900)
Supplement: Supplemental material [file TNZR_A_2251900_SM4890.docx]

ONLINE SUPPLEMENTARY DATA FOR JRSN:

**A cross-sectional online survey of depression symptoms among New Zealand’s Asian community in the first 10 months of the COVID-19 pandemic**

Richard J. Siegert^1^*, Andrew Zhu^2^, Xiaoyun Jia^3^, Guanyu Jason Ran^4^, Nigel French^5^, David Johnston^6^, Jun Lu^7^, Liangni Sally Liu^8^*

1. **Mean and standard deviations of total 10-item CESD score for sample by country of origin.**

| **Report** | | | |
| --- | --- | --- | --- |
| CESD10 | | | |
| D1: What is your country of origin? | Mean | N | Std. Deviation |
| Mainland China | 10.0574 | 126 | 4.62390 |
| Hong Kong | 8.3798 | 8 | 3.29009 |
| Taiwan | 5.4261 | 5 | 4.52847 |
| Vietnam | 13.6876 | 6 | 10.04160 |
| Cambodia | 11.4880 | 5 | 8.34542 |
| India | 12.6032 | 135 | 6.47857 |
| The Philippines | 9.3797 | 41 | 5.88656 |
| Korea | 12.7554 | 20 | 5.24188 |
| Japan | 10.1692 | 10 | 6.59596 |
| Sri Lanka | 12.2242 | 9 | 5.99803 |
| Singapore | 10.0364 | 8 | 7.79665 |
| Malaysia | 9.1592 | 19 | 7.05151 |
| Thailand | 14.3990 | 4 | 8.69521 |
| Other Asian countries | 14.8740 | 5 | 3.24260 |
| Total | 11.0713 | 402 | 6.02151 |

1. **Mean and SD of total 10 item CES-D score by geographical region.**

| **Report** | | | |
| --- | --- | --- | --- |
| Total_CESD_10 | | | |
| D2: Where do you currently live in New Zealand? | Mean | N | Std. Deviation |
| Northland Region | 15.2481 | 6 | 6.74630 |
| Auckland Region | 11.1959 | 262 | 6.00868 |
| Waikato Region - Hamilton | 9.9991 | 15 | 3.92871 |
| Waikato Region - other | 5.4775 | 1 | . |
| Bay of Plenty Region | 10.8962 | 6 | 7.24244 |
| Hawke's Bay Region | 9.2187 | 3 | 4.62891 |
| Taranaki Region | 6.8506 | 4 | 4.35612 |
| Manawatu-Wanganui Region | 10.5819 | 9 | 7.64817 |
| Wellington Region | 10.4962 | 52 | 6.61070 |
| Tasman Region | 12.0000 | 1 | .00000 |
| Marlborough Region | 16.0000 | 2 | .00000 |
| Canterbury Region - Christchurch | 11.0154 | 28 | 5.81510 |
| Canterbury Region - other | 10.7222 | 2 | 3.05396 |
| Otago Region - Dunedin | 9.6806 | 3 | 7.65534 |
| Otago Region - Queenstown | 9.8502 | 2 | 5.63609 |
| Otago Region - other | 18.0000 | 1 | .00000 |
| Southland Region | 13.6079 | 4 | 5.91820 |
| Total | 11.0713 | 402 | 6.02151 |
